# Genetic testing for oral clefts: reflections based on a single Brazilian public genetics service

**DOI:** 10.1186/s13023-025-03967-y

**Published:** 2025-08-16

**Authors:** Tamiris Nogueira Bezerra Bueno, Társis Paiva Vieira, Vera Lúcia Gil-da-Silva-Lopes

**Affiliations:** Department of Medical Genetics and Genomic Medicine, University of Campinas (Unicamp), Tessália Vieira de Camargo Street, 126, Campinas, 13083-887 SP Brazil

**Keywords:** Cleft lip, Cleft palate, Diagnosis, Public health, Genomics, Whole exome sequencing, Chromosomal microarray analysis

## Abstract

**Background:**

Genomic medicine has allowed for an improvement in the diagnosis and molecular understanding of congenital defects. However, its implementation into routine clinical practice demands enormous challenges worldwide. This study describes the etiological diversity and access to genetic diagnosis of individuals with oral clefts (OC) at a single genetics service.

**Results:**

This cross-sectional and descriptive study analyzed primary records of the Brazilian Database on Craniofacial Anomalies from 2006 to 2019, before the National Policy of Comprehensive Care for People with Rare Diseases (NPCCPRD) implementation in this service. Among 103 individuals (51 Female and 52 Male), the proportion of syndromic OC (SOC) and non-syndromic OC (NSOC) was 73.8% and 26.2%, respectively, showing that NSOC seems not to be referred for genetic evaluation. Diagnosis occurred in 64/103 (62.13%) cases; 36/64 (56,25%) had clinical diagnoses, of which 27/36 were NSOC. The tests allowing a conclusive diagnosis were whole exome sequencing (WES) (11/20–55.00%), followed by chromosomal microarray analysis (CMA) (4/52–7.69%), Fluorescent in situ hybridization (FISH) (6/21–28.57%), multiplex ligation-dependent probe amplification (MLPA) (2/32–6.25%), and G-banding karyotype (6/72–8.33%). Age at diagnosis ranged from 0 to 46 years (mean = 9.56 /median = 7). Diagnostic investigations of 39/76 SOC cases are still ongoing, relying on clinical follow-up and genetic tests.

**Conclusions:**

Etiological diversity reinforces the need for different laboratory resources and clinical follow-up. These results and reflections about the need to implement Genomic Medicine are of universal interest. They also show the need to improve public health policies for genetic evaluation, diagnostic tests, and genetic counseling for an effective NPCCPRD.

## Background

Technological advances in genetic diagnosis have been significant, providing a deeper understanding of the genetic basis of several rare diseases. Methods such as next-generation sequencing (NGS) have revolutionized how we identify and understand genetic diseases. However, the integration of these technologies into clinical practice faces significant challenges. Therefore, despite promising advances, the full integration of genetic technologies into routine clinical practice still requires overcoming several challenges [1].

Craniofacial anomalies (CFA) are a heterogeneous group with extensive nosology, which may include cranial and/or facial structure alterations. Furthermore, CFA can be isolated or associated with other morphological defects, increasing complexity and clinical management. Additionally, CFA treatment is multidisciplinary, prolonged, and specialized, becoming a public health problem [2].

Among CFA, typical oral clefts (TOC) are the most prevalent and one of the most common congenital defects in humans. The estimated prevalence is 1 in 700 live births, varying by geographical region and ethnicity [2, 3]. Regarding the association with other congenital defects, non-syndromic oral clefts (NSOC) represent 70% of cases, and the remaining 30% are syndromic oral clefts (SOC) [3].

It is also worth noting that differentiating between NSOC and SOC can be challenging from a clinical perspective, as there may be subtle clinical signs, and families with SOC can go unnoticed by non-specialized professionals [4, 5].

In Brazil, public healthcare policies regulate craniofacial anomalies’ treatment and genetic evaluation. Since 1994, the Ministry of Health has supported multidisciplinary treatment for orofacial clefts. Since 2014, Ordinance No. 199/2014 has established the National Policy for Comprehensive Care for People with Rare Diseases (NPCCPRD), providing guidelines for genetic diagnosis and counseling within the Unified Health System (UHS). Despite these regulations, challenges persist in ensuring widespread access to advanced diagnostic techniques [6–10]. Identifying the population’s needs can support the implementation of regional and national proposals within the scope of the NPCCPRD.

This study aimed to characterize access to clinical investigation and genetic testing for individuals with OC treated at the Clinical Genetics Service of HC-Unicamp before the implementation of the NPCCPRD in this hospital.

The approach in a single public genetics service comprehensively shows a universal issue: the difficulties of implementing genomic medicine for OC and brings insights into other genetic disorders. It helps improve public health policies for genetic evaluation, diagnostic tests, and genetic counseling.

## Methodology

The Hospital de Clínicas of the University of Campinas (HC-Unicamp) was designated by the Ministry of Health as a Reference Center for Rare Diseases in December 2019 through Ordinance No. 199, dated January 30, 2014. The Craniofacial Dysmorphology Outpatient Clinic (CDOC) has been active since 2002 and attends to intern patients referred from the regional public health system.

Due to clinical and etiological heterogeneity of OC, the regular clinical investigation process followed the anamnesis and clinical-dysmorphological assessment. When indicated, the G-banding karyotype - the only one available in the public service at the time - was conducted. In addition to clinical care through UHS, patient registration in the Brazilian Database on Craniofacial Anomalies (BDCA) allows voluntary participation in research projects with complementary genetic tests, accepted by approximately 80% of the patients (unpublished data). The BDCA is one of the voluntary initiatives of Brazil’s Craniofacial Project (BCFP), which aims to produce scientific evidence to support public policies in craniofacial genetics [11–13].

Therefore, it is a cross-sectional and descriptive study evaluating primary data from individuals with OC registered in the BDCA by the CDOC. The chosen period was from August 2006 to December 2019, before the implementation of the NPCCPRD.

The diagnostic tests previously inserted in BDCA are results from other BCFP projects and were compiled for this study (tests were not reanalyzed). Depending on the diagnostic hypothesis, and the inclusion criteria in different research projects, these included G-banding karyotype, FISH, with the TUPLE1 probe (Cytocell Aquarius^®^ or Visys Abbott©), MLPA, with the P250 kit (MRC-Holland^®^, Amsterdam, the Netherlands), CMA trough Cytoscan 750 K or Cytoscan HD (Affymetrix ^®^), and Whole Exome Sequencing (WES) (Agilent SureSelect XT Human All Exon Kit V6 (Agilent Technologies^®^, Santa Clara, CA, USA). The latter was also performed on individuals without a previously defined diagnosis, with DNA samples available in the biorepository of the BDCA.

### DNA samples

Genomic DNA samples stored in the BDCA biorepository were obtained from peripheral blood samples using phenol/chloroform extraction, following a standard protocol at the Human Cytogenetics and Cytogenomics Laboratory of the School of Medical Sciences/UNICAMP. All samples were purified by Microcon-30 kDa Centrifugal Filter Unit with Ultracel-30 membrane (Millipore, Billerica, MA, USA).

### Whole exome sequencing (WES)

The present study performed WES through the Illumina NovaSeq 6000 Sequencing System (Illumina, Inc©, San Diego, CA, USA) to generate paired-end, 2 × 150 bp reads with on-target 140x coverage. The raw data was extracted in Fastq format, and analysis and classification of Variants used Varstation© v.2.0. The variant filtering was performed according to the following criteria: minimum coverage of 10x; variant allele frequency (VAF) ≥ 0.2; variant consequence including missense, nonsense, frameshift, changes in splicing sites, non-frameshift indels, start loss, stop loss, and synonymous variants; allelic frequency < 0.5% in GnomAD, ExAC, and ABraOM.

The variants were classified according to the recommendations of the American College of Medical Genetics (ACMG) guidelines [14]. The variants were further analyzed using public databases (ClinVar (https://www.ncbi.nlm.nih.gov/clinvar/) and Varsome (https://varsome.com/)) and a review of the literature. After these procedures, cases were clinically reviewed with a follow-up consultation when possible.

## Results

The cohort consisted of 103 individuals (52 males and 51 females). The age at first assessment ranged from 0 to 52 years (mean = 6.9 / median = 2).

Regarding association with other defects, 76/103 represented SOC (73.7%), and 27/103 were NSOC (26.2%). Among cases initially classified as suspected NSOC, the hypothesis was modified to SOC in 10 individuals based on clinical evolution. So far, a diagnostic conclusion has occurred in 64/103 cases (62.1%).

### Cases with NSOC

Of the 27/103 individuals diagnosed with NSOC, the diagnosis was based on clinical evaluation; however, in 18 cases, karyotyping was performed, and no alterations were detected. The average age at the first consultation was 5.7 years (minimum = 0, maximum = 39, median = 0), and the age at diagnosis ranged from 0 to 39 years (mean = 6.5, median = 1).

### Cases with SOC

Regarding the 76 cases evaluated with suspected SOC, 37 had a diagnosis conclusion. The age at diagnosis ranged from 0 to 46 years (mean = 9.5 and median = 7). The diagnosis was clinical in 9/37 (24.3%) and 28/37 (75.6%) through genetic testing: six cases by karyotype testing and the remaining 22 using different laboratory techniques through research projects. In one individual, there was a concomitance of alterations (17p12 duplication, variants of uncertain significance in compound heterozygosity in the *PRX* gene, and a likely pathogenic variant in heterozygosity in the *MECP2* gene). In another case, the phenotype and clinical evolution were compatible with detected genomic findings (Holoprosencephaly and Nance-Horan syndrome).

The remaining 39/103 cases (37.8%) did not have a conclusive diagnosis despite performing karyotyping for 25 individuals, FISH for four, MLPA for 18, CMA for 25, and WES for nine individuals. Some of them are awaiting clinical follow-up, while others are waiting for the possibility of new tests.

Table 1 summarizes the nosology of individuals investigated for suspected SOC.

**Table 1:** Nosology of individuals investigated for suspected SOC (*n* = 76)

Diagnosis		OMIM	Number of cases
Clinical	Van der Woude Syndrome	#119,300	2
	Treacher Collins Syndrome	#154,500	1
	Gorlin Syndrome	#109,400	1
	Moebius – Poland		1
	Waardenburg Syndrome	#148,820	1
	Apert Syndrome	#101,200	1
	Aarskog Syndrome	#305,400	1
	Stickler Syndrome	#108,300	1
	**Total**		**9**
Chromosomal abnormalities/ Genomic Imbalances	47,XX,+13/46,XX		1
	46,XX, del(18)(q21)		1
	45,XX, der(15;18)(q10;q10)		1
	46,XY, rec(18)dup(18q)inv(18)(p11.2q21.3)mat		1
	46,XY, add(10)(q26)		1
	47,XXY(Klinefelter Syndrome)		1
	ish del(7)(q11.23q11.23)(ELN-) (Williams Syndrome)	# 194,050	1
	22q11.2 proximal deletion (22q11.2 deletion syndrome)	# 188,400	6
	arr[GRCh38] 17q12(36375365_37925364)x3		1
	arr[GRCh37] 2q21.2q24.1(132613883_158465121)x3		1
	arr[GRCh37] 17p13.3(525_2101504)x1 (Miller-Dieker Syndrome)	#247,200	1
	46,XY, ish del(4)(p16.3) (Wolf-Hirschhorn Syndrome)	#194,190	1
	arr[GRCh37] 17p12(14087933_15436894)x3 (Charcot -Marie -Tooth tipo 1 A)**	#118,220	1
	**Total**		**18**
Monogenic disorders	*TP63*:c.1813 C > T:p.(Arg605Trp) (ADULT Syndrome)	#103,285	1
	*SOX9*:c.508 C > G:p.(Pro170Ala) (Campomelic Dysplasia)	# 114,290	1
	Likely pathogenic variant: *SETX*:c.6996_7002del: p.(Asp2332Glufs*11)	Need follow-up	1
	Pathogenic variant: *ANKRD11*:c.4395_4396del: p.(His1465fs) (KBG Syndrome)	# 148,050	1
	Pathogenic variant: *SATB2*:c.715 C > T - p.(Arg239*) (SATB2 Syndrome)	# 612,313	1
	Likely pathogenic variant: *TGFBR1*:c.1025 A > G; p.(Asn342Ser) (Loyes-Dietz Syndrome)	# 609,192	1
	*CDH1*: c.760 G > A p.(Asp254Asn) (Blepharocheilodontic Syndrome)	#119,580	1
	*NHS*:c.2731G > T:p.(Glu911Ter) / Nance-Horan Syndrome/ Holoprocencephaly *SHH*:c.437G > A:p.(Trp146Ter)#	#302,350/ #142,945	1
	*SOX11*:c.251G > A:p.(Gly84Asp)	Awating reevaluation	1
	*MYO18B*:c.7597 C > T:p.(Arg2533*) / *MYO18B*:c.510_511del: p.(His171fs)	Awating reevaluation	1
	*MECP2*:c.763 C > T:p.(Arg255Ter)** Ret Syndrome PRX: c.892 C > T p.(Pro298Ser)/PRX: c.133 C > G p.Arg45Gly** Charcot-Marie-Tooth Desease 4 F	#312,750 / #614,895	1
	**Total**		**11**
Undiagnosed			**39**
TOTAL			**76**

**, #: Same individual with concomitance of diagnoses

### A comprehensive evaluation of the cohort

In total, 36 individuals received a clinical diagnosis, with 27 having NSOC and 9 with SOC. After a genetic test, a conclusive diagnosis was achieved for 28 individuals: six with chromosomal anomalies detected by G-banding karyotype, 12 with genomic imbalances detected by FISH, MLPA, and CMA, and 11 with monogenic disorders concluded by WES. Two individuals had more than one laboratory alteration identified. In one, the indication for WES occurred after the detected genomic imbalance did not justify the clinical picture during the dysmorphological reassessment.

**Fig. 1:**
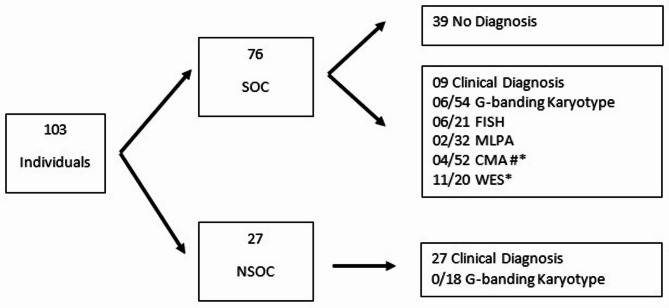
Schematic design representing the evaluation/genetic test for diagnostic conclusion according to clinical suspicion in this cohort. SOC: Syndromic Oral Cleft; NSOC: non-syndromic oral cleft. *One individual had different diagnoses determined by CMA and WES. # one individual underwent more than one test in a research context

The genetic test with higher diagnostic yield in this sample was WES (11/20–55.00%), followed by CMA (4/52–7.69%), FISH (6/21–28.57%), MLPA (2/32–6.25%), and karyotype (6/72–8.33%). Figure 1 shows the evaluation/genetic test that led to the diagnostic conclusion in this cohort.

## Discussion

Considering the burden of OC on affected individuals and their families, access to etiological investigation, early diagnosis, and genetic counseling is important. These procedures are essential for evaluating risks for future generations and minimizing impacts on the health of affected individuals [15].

In the present study, the average age at first assessment for clinical genetics evaluation was 6 years and 9 months. A national study of access to genetic tests involving 1463 Brazilian individuals with OC shows an average age of 8 years and 5 months for genetic testing among SOC. The present case series is part of the cohort analyzed in the cited article [12].

Additionally, the proportions of individuals with NSOC (26.2%) and SOC (73.8%) were very different from the proportions of syndromic and non-syndromic OC cases described in the literature, with a predominance of NSOC (70%) [3]. This discrepancy may be related to a lack of referral of individuals with NSOC for genetic evaluation, with a bias of referring only individuals with multiple congenital anomalies for specialized evaluation.

However, some previous studies reported a similar proportion of SOC in other populations. In a study conducted with 1000 patients with OC in an American center for craniofacial disorders, 63.4% of the cases had other associated anomalies [16]. Two other Brazilian studies, one with patients from the clinical genetics service and another with patients from the genetics and otorhinolaryngology outpatient clinic, also reported a higher proportion of SOC cases, with 59.6% and 68.6%, respectively [10, 17].

Clinical follow-up of 10 cases previously classified as NSOC among the individuals in the present study led to the diagnosis of SOC. It is expected for some cases, as further investigation or clinical evolution can reveal other congenital defects, developmental delays, or intellectual deficits. This aspect reinforces the need for longitudinal follow-up and phenotype reassessment. It highlights the complexity of differentiating between the two groups to ensure accurate diagnosis, genetic counseling, and appropriate treatment [8, 9].

In some cases, such as NSOC, clinical evaluation may be sufficient to establish the diagnosis, especially when the symptoms are characteristic and do not raise suspicions of a syndromic phenotype. Among the individuals in this case series, a diagnostic conclusion based on clinical evaluation was possible in 27 NSOC and nine SOC. However, microforms of OC, which may include discontinuity of the *orbicularis oris* muscle, dental hypoplasia, or agenesis, have been recognized over time. With these new pieces of information, individuals with microforms may not have been detected in this sample. Therefore, while the clinical evidence used may have been sufficient to indicate the diagnosis, it may not have been appropriate for genetic counseling [18].

Recent studies showed that in families with vertical transmission, it is possible to find causative genes segregating as an autosomal dominant trait [18, 19]. Therefore, a more detailed investigation of parents - including dental inspection and ultrasound of the *orbicularis oris* muscle - could modify the proportion of affected individuals in families and, in some situations, indicate the need for WES investigation.

G-banding karyotype, the only widely available genetic test through the UHS, was sufficient for diagnostic conclusion and genetic counseling in 6/72 (8.33%) cases in which karyotyping was performed. Another Brazilian study of 115 individuals with OC reported that the G-banding karyotype detected chromosomal abnormalities in seven cases (6.0%) [10]. Considering the other cases with etiological diagnosis, 4/28 (14.2%) had genomic imbalances detected by CMA, and 11/28 (39.2%) had monogenic disorders revealed by WES, the importance and benefits of including CMA and WES application become evident [20].

Two specific individuals exemplify the challenges of SOC diagnosis and reinforce the need for clinical genetic follow-up when the association of a variant with the phenotype is unclear.

In the first one, a 17p12 duplication was identified by CMA (arr[GRCh37] 17p12(14087933_15436894)x3), leading to the diagnosis of Charcot-Marie-Tooth type 1 A syndrome (# 118220). However, after clinical evaluation, this genetic alteration was not considered to be associated with the entire clinical picture presented by the individual, prompting further investigation with WES. The latter revealed a likely pathogenic variant in the *MECP2* gene (MECP2: c.763 C > T(p.Arg255Ter)), which, combined with the evolving phenotype, allowed a diagnostic conclusion. Additionally, VUSses were detected in heterozygosity in the *PRX* gene, responsible for Charcot-Marie-Tooth 4 F syndrome (# 614895), which may have some influence on the phenotype. The literature has reported cases where more than one variant is present, influencing the clinical picture of individuals [21]. Despite these findings, the oral cleft seems to be a random association.

The second individual had a conclusive diagnosis after WES. Reverse phenotyping detected a mild clinical finding of Holoprosencephaly type 3 (OMIM #142945) and features related to Nance-Horan Syndrome (OMIM #302350).

Considering the 20 individuals in this case series who underwent WES, a diagnostic conclusion was possible in 11, corresponding to 55%. Studies addressing the diagnostic yield by WES in SOC are rare, varying from 16,7% in OC [22] to 35,27% in OC associated with microphthalmia-anophthalmia-coloboma [23]. Also, other studies highlight the importance of WES in diagnosing other groups of patients with suspicion of a genetic disorder, such as neurodevelopmental disorders, with a diagnostic rate ranging from 30 to 57% [24]. The results herein emphasize the need to expand access to genomic testing for individuals with SOC as a specific population in UHS.

Despite being a common etiology for SOC [3], this study did not diagnose teratogenic causes. Additionally, 39/76 individuals classified with SOC did not have a conclusive diagnosis. The lack of available tests for all cases also directly impacts the number of individuals without a diagnosis, as they depend on research projects with specific inclusion criteria. On the other hand, clinical follow-up and literature review for new insights are usual approaches to achieving a diagnosis [9].

In the present study, WES allowed the highest diagnostic yield, followed by CMA, FISH, MLPA, and karyotype. Considering the applications of WES, which in some clinical contexts also allow Copy Number Variation (CNV) analysis and diagnosis, there is scientific support for considering it as the first-choice diagnostic test for individuals with SOC, except in those with an evident phenotype of numeric chromosomal abnormality. However, depending on the purpose of the diagnosis, this strategy may not be as suitable for investigating parents for genetic counseling. In this situation, other complementary laboratory techniques may be necessary.

Among the 20 individuals who underwent WES, 9 remained undiagnosed. Data reanalysis should occur at least every two years, as knowledge evolution may lead to variant reclassification and diagnostic conclusion [25]. Family follow-up and the allocation of human resources for data reanalysis are necessary to apply this strategy. These variables should be considered when establishing a public policy for this population group.

The etiological diversity in the group of individuals reinforces the need for prior clinical-dysmorphological assessment before genetic testing, access to different diagnostic techniques, and reassessment when tests are inconclusive to understand the natural history and continue the investigation.

The wide range of age at etiological diagnosis presented in this study (from 0 to 46 years) may be directly related to the availability of novel genetic tests. Technological advances and increased access to complementary tests, such as CMA and WES, carried out during the study period through research projects, allowed for early diagnosis in cases that would have remained undiagnosed through the UHS.

The integration of genomics into medical practice has the potential to transform the approach to diagnosing and managing conditions like OC, offering a structured framework that enhances patient care. This process can include four main stages: phenotypic assessment and generation of prior risk, evaluation of the clinical utility of genomic testing, analysis and interpretation of genomic variations, and patient management based on genomic information [26]. Based upon this, the BCFP has been acting in the first three steps, providing evidence for a comprehensive evaluation of the clinical and genetic profile of patients with OC in Brazil [11, 23, 27, 28]. It can serve as a foundation to improve access to accurate diagnoses and personalized interventions, addressing the unique challenges of this population group.

This study reflects the reality of a public service that, through research, has contributed to the diagnosis and genetic counseling of individuals with SOC and reinforces the need for a complete dysmorphological evaluation seeking OC microforms. This example, supported by scientific literature, highlights the need to develop public health policies to improve the access of individuals with OC to genetic evaluation and testing. Accurate diagnostic access, genetic counseling, and appropriate clinical follow-up would positively impact this population group’s health and quality of life. It also improves knowledge about natural history and comorbidities related to SOC, facilitating multiprofessional follow-up and economic cost planning [29]. Therefore, this approach should be universally considered.

## Data Availability

The datasets generated and/or analysed during the current study are not publicly. available because it contains information from patients whose consent was given for specific use.

## Abbreviations

CFA: Craniofacial anomalies
TOC: typical oral clefts
RRTDFC: Reference Network for Craniofacial Treatment
UHS: Unified Health System
NPCCPRD: National Policy of Comprehensive Care for People with Rare Diseases
ICD: International Classification of Diseases
SOC: Syndromic oral clefts
NSOC: Non-syndromic oral clefts
FISH: Fluorescence in situ Hybridization
CMA: Chromosomal Microarray Analysis
MLPA: Multiplex Ligation-dependent Probe Amplification
NGS: Next-Generation Sequencing
BDCA: Brazilian Craniofacial Anomalies Database
BCFP: Brazil’s; Craniofacial Project
CDOC: Craniofacial Dysmorphology Outpatient Clinic
Unicamp: Universidade Estadual de Campinas
HC: Hospital de Clínicas
WES: Whole Exome Sequencing
OC: Oral clefts
CNV: Copy Number Variation
ACMG: American College of Medical Genetics and Genomics
